# Skull bone erosion in a silent atypical meningioma: A case report and literature review

**DOI:** 10.1016/j.ijscr.2025.111851

**Published:** 2025-08-25

**Authors:** Omar Sawafta, Jehad Khamaysa, Dawoud Hamdan, Orabi Hajjeh, Jehad M.J. Zeidalkilani, Abdelrahman Abuali

**Affiliations:** a Department of Medicine, An Najah National University, Nablus, Palestine; b Department of Radiology, Tubas Turkish Governmental Hospital, Tubas, Palestine; c Department of Internal Medicine, MercyOne Siouxland Medical Center, Sioux City, IA, USA

**Keywords:** Atypical meningioma, Skull bone erosion, Extra-axial tumor, Asymptomatic presentation

## Abstract

**Introduction and importance:**

Meningiomas are the most common primary tumors of the central nervous system. Atypical meningiomas, classified as World Health Organization (WHO) grade II, are relatively rare, accounting for 5–7 % of cases, and are known for their aggressive behaviour, including higher recurrence rates and potential brain invasion. Early detection and intervention are crucial, even in asymptomatic patients.

**Case presentation:**

We report a case of a 45-year-old male presenting with a two-year history of painless, progressive swelling over the left parietal region. Neurological examination was normal. Imaging revealed a well-defined extra-axial lesion with bone erosion suggestive of atypical meningioma. Surgical resection via craniotomy was performed, and histopathology confirmed a WHO grade II atypical meningioma. The postoperative course was uneventful, and the patient remained asymptomatic with no recurrence on follow-up imaging.

**Clinical discussion:**

Although often asymptomatic, atypical meningiomas can exhibit invasive features requiring prompt diagnosis and surgical management. MRI findings and histological markers, including mitotic activity and focal brain invasion, aid in accurate grading. Close follow-up is recommended due to the higher recurrence potential compared to benign subtypes.

**Conclusion:**

This case highlights the importance of considering atypical meningioma in patients with isolated skull swelling. Timely diagnosis and surgical management are essential to prevent complications and ensure a favorable prognosis.

## Highlights

• Rare Asymptomatic Presentation: A WHO Grade II atypical meningioma presented as a painless parietal skull swelling *without neurological deficits*.
• Imaging revealed skull bone erosiona key feature distinguishing atypical from benign meningiomas—highlighting the role of CT/MRI in early detection.
• Collaboration between radiology, neurosurgery, and pathology ensured accurate diagnosis (histopathology: ≥4 mitoses/10 HPF) and successful gross total resection.
• Surgical Success: Craniotomy achieved complete tumor excision with no postoperative complications or recurrence at follow-up.
• Clinical Significance: This case underscores the importance of considering meningiomas in isolated skull deformities, even in asymptomatic patients, to prevent delayed intervention.

## 1 Introduction

Meningiomas are the most common primary tumors of the central nervous system, arising from arachnoid cap cells of the meninges. They occur more frequently in females, with a male-to-female ratio of 2:3. According to World Health Organization (WHO) classification, meningiomas are divided into Grade I (benign), Grade II (atypical), and Grade III (anaplastic), with atypical meningiomas accounting for 5–7 % of cases [1]. Grade II tumors exhibit more aggressive behaviour, including a higher recurrence rate and brain invasion. Histologically, atypical meningiomas are defined by ≥4 mitoses per 10 HPF or specific architectural and cytological features. On MRI, they present as heterogeneously enhancing, extra-axial masses with broad dural attachment and surrounding edema. Management involves surgical resection, with adjuvant radiotherapy considered in selected cases [2].

We presented a case of a 45-year-old male with a two-year history of painless, progressive left parietal scalp bulging. Imaging revealed an extra-axial lesion with skull erosion consistent with atypical meningioma. Surgical resection confirmed WHO grade II meningioma. The patient remained asymptomatic postoperatively with no evidence of recurrence.

## 2 Case presentation

A 45-year-old male with no significant past medical history presented to the outpatient clinic with a chief complaint of a painless, progressive bulging over the left side of his head. The swelling had been gradually enlarging over approximately two years. The patient denied any history of trauma, headache, seizures, visual disturbances, focal neurological deficits, or constitutional symptoms. His medical, surgical, and family histories were unremarkable, and he was not on any regular medications. He reported no functional limitations or recent changes in his general health status.

On physical examination, a firm, non-tender, immobile mass was palpable over the left parietal region of the skull. The overlying skin appeared normal, with no signs of discoloration, ulceration, or inflammation. Neurological examination was completely normal, with intact cranial nerves, full motor strength, preserved sensation, normal reflexes, and no evidence of cerebellar dysfunction or gait disturbance.

**Fig. 1:**
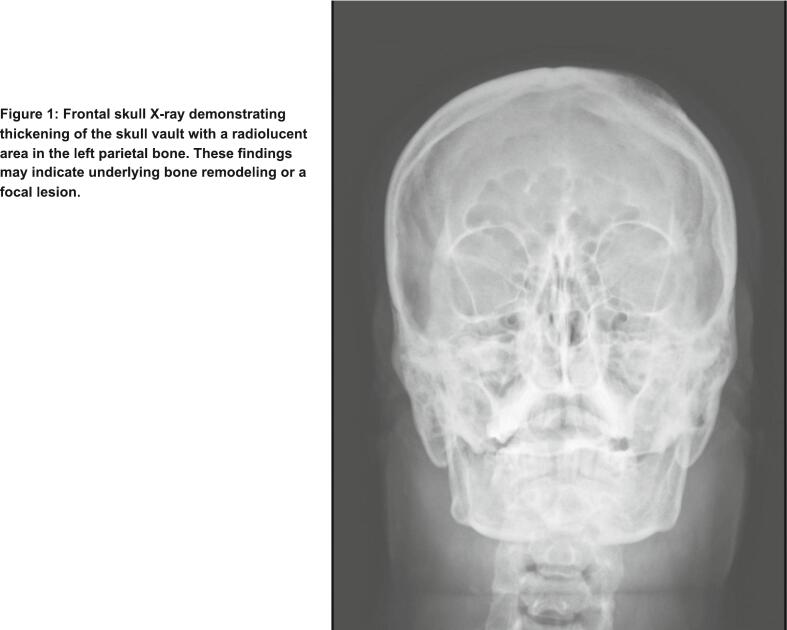
Frontal skull X-ray demonstrating thickening of the skull vault with a radiolucent area in the left parietal bone. These findings may indicate underlying bone remodeling or a focal lesion.

**Fig. 2:**
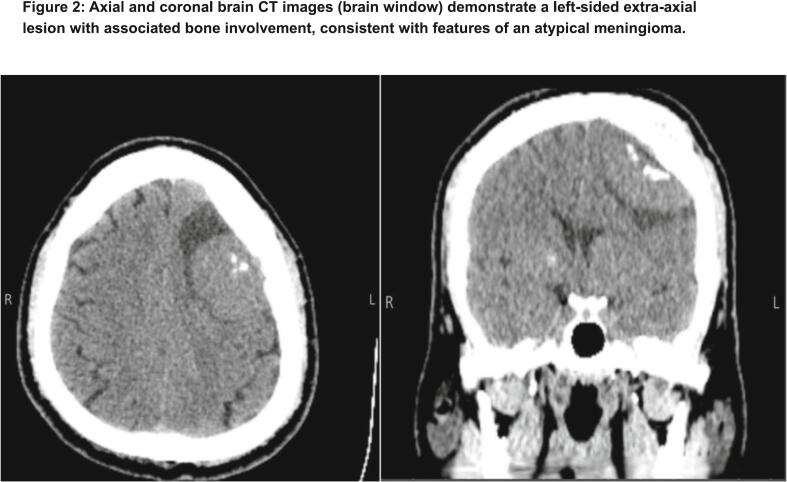
Axial and coronal brain CT images (brain window) demonstrate a left-sided extra-axial lesion with associated bone involvement, consistent with features of an atypical meningioma.

**Fig. 3:**
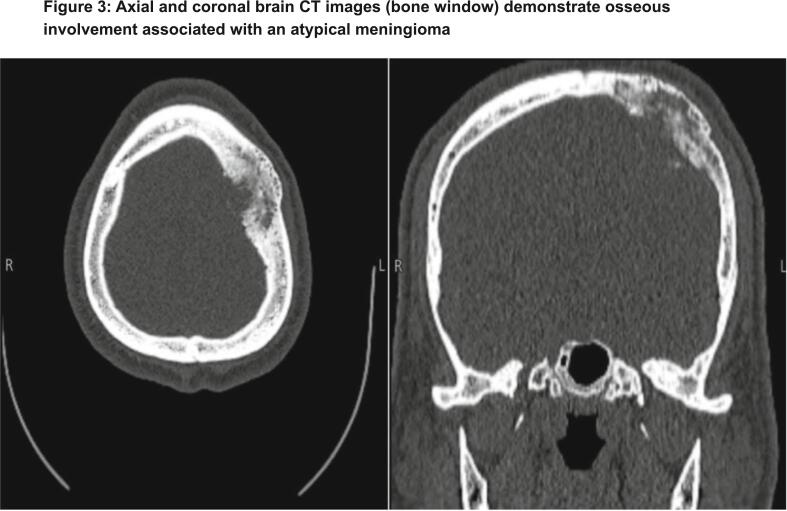
Axial and coronal brain CT images (bone window) demonstrate osseous involvement associated with an atypical meningioma.

Initial imaging with a frontal skull X-ray revealed thickening of the skull vault and a radiolucent area within the left parietal bone (Fig. 1). These findings raised suspicion for an underlying bone lesion or remodeling process. A non-contrast brain computed tomography (CT) scan was subsequently performed demonstrated a well-defined, left-sided extra-axial lesion with associated osseous involvement, consistent with features of an atypical meningioma (Fig. 2). Additional CT images in the bone window confirmed localized bone changes in the affected area (Fig. 3).

**Fig. 4:**
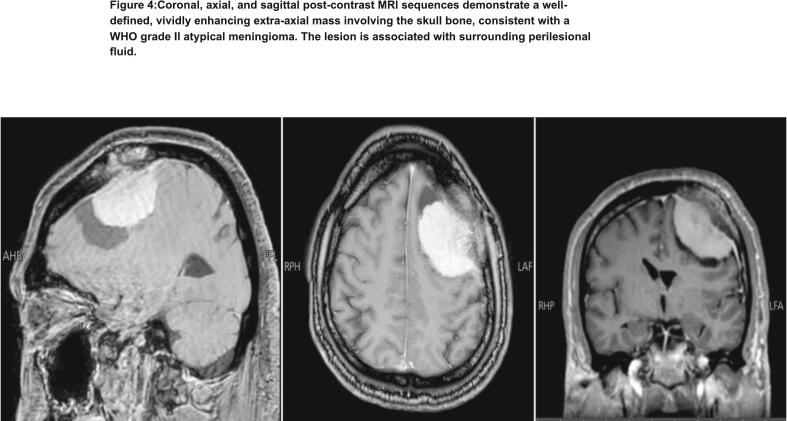
Coronal, axial, and sagittal post-contrast MRI sequences demonstrate a welldefined, vividly enhancing extra-axial mass involving the skull bone, consistent with a WHO grade II atypical meningioma. The lesion is associated with surrounding perilesional fluid.

Further evaluation with post-contrast magnetic resonance imaging (MRI) of the brain was undertaken shows well-demarcated extra-axial mass involving the left parietal bone. The lesion demonstrated radiologic characteristics consistent with a WHO grade II atypical meningioma and was associated with mild perilesional fluid, but without evidence of brain parenchymal invasion or significant mass effect (Fig. 4).

The patient was referred to the neurosurgery department for definitive management. Following multidisciplinary discussion, surgical resection was advised. A craniotomy was performed, and gross total excision of the lesion was achieved without intraoperative complications. Histopathological analysis confirmed the diagnosis of WHO grade II atypical meningioma, showing increased mitotic activity and focal brain invasion, without necrosis.

The postoperative course was uneventful. The patient was discharged in stable condition with no new neurological deficits. Regular clinical and radiologic follow-up was arranged. At the most recent follow-up, the patient remained asymptomatic, and surveillance imaging showed no signs of recurrence.

## 3 Discussion

Meningiomas are the most common primary tumors that arise in the central nervous system (CNS) and originate from the meninges, most commonly from the arachnoid cap cells [1]. This condition is diagnosed in males and females at a ratio of 2 to 3 [2]. According to the WHO classification, meningiomas are categorized into three grades: Grade I (benign), accounting for approximately 90 % of cases; Grade II (atypical), comprising about 5–7 %; and Grade III (anaplastic or malignant), representing 1–3 % of all meningiomas [3]. The latter often have poor prognosis, warranting effective screening and management strategies [1].

Grade II meningiomas are subclassified into atypical, clear cell and chordoid meningiomas. With atypical meningiomas being the most prevalent subtype. They are defined by one or more of the following histopathological features: a mitotic index of ≥4 mitoses per 10 high-power fields (HPF), evidence of brain invasion, or the presence of three or more of the following criteria: sheet-like growth pattern, hypercellularity, prominent nucleoli, small cells with a high nuclear-to-cytoplasmic (N:C) ratio, and spontaneous foci of necrosis [4].

Atypical meningiomas are considered more aggressive than benign variants, with a five-year recurrence rate of approximately 41 % [5]. Common genetic changes include loss of the NF2 gene, genomic instability, and mutations in SMARCB1 which often show a hypermethylated phenotype and upregulation of PRC2 complex components [6]. Patients often present with headache, seizures, confusion, drowsiness, in addition to signs of increased intracranial pressure, such as projectile vomiting [5].

On Magnetic Resonance imaging (MRI), they typically appear as lobulated, well-circumscribed extra-axial masses with a broad dural attachment [7]. They show heterogeneous enhancement and are associated with a greater degree of surrounding vasogenic edema compared to benign meningiomas [8].

Observation and surgical resection remain the primary treatments for meningiomas. For atypical meningiomas, adjuvant radiotherapy is often evaluated on a case-by-case basis, whereas it is generally recommended for anaplastic meningiomas due to their aggressive nature [9]. In adults, prognosis is influenced largely by age, with younger patients generally experiencing better outcomes. For atypical meningiomas, routine MRI surveillance is recommended every 6 to 12 months during the first five years following treatment, and then every two years thereafter [5].

In the present case, the patient's asymptomatic skull swelling and imaging findings (e.g., bone erosion on CT, heterogeneous enhancement on MRI) aligned with typical features of atypical meningiomas, as described in the literature. However, the absence of neurological deficits despite focal brain invasion on histopathology underscores the unpredictable behaviour of WHO Grade II lesions. This discrepancy between indolent clinical presentation and aggressive histologic features reinforces the need for vigilant surveillance, even in seemingly low-risk cases. Our management approach—prioritizing gross total resection without adjuvant radiotherapy—reflects the ongoing debate about optimal treatment for atypical meningiomas, particularly when complete excision is achieved. The favorable outcome here supports surgical intervention as a cornerstone for localized lesions, though long-term follow-up remains essential given the reported 41 % five-year recurrence rate.

## 4 Conclusion

This case emphasizes the importance of considering atypical meningioma in patients presenting with asymptomatic skull deformities. Despite its indolent clinical course, imaging and histology revealed an aggressive lesion requiring surgical intervention. Early detection and timely management are essential to prevent complications and ensure favorable long-term outcomes in atypical meningioma.

## 5 Method

The work has been reported in line with the SCARE criteria [10].

## Informed consent

Written informed consent was obtained from the patients for their anonymized information to be published in this article.

## Ethical approval

Our institution, An-Najah National University, does not require ethical approval for reporting individual cases or case series.

## Funding

No specific grant from funding agencies was received for this work.

## Author contribution

**Omar Sawafta** contributed to data curation and writing the original draft. **Jehad Khamaysa** handled radiological analysis and visualization. **Dawoud Hamdan** assisted in clinical data collection and writing the original draft. **Orabi Hajjeh** (corresponding author) oversaw conceptualization, supervision, and writing – review & editing. **Jehad M.J. Zeidalkilani** contributed to literature review and writing – review & editing. **Abdelrahman Abuali** validated pathological findings and edited the manuscript. All authors read and approved the final manuscript, agreeing to take full responsibility for all aspects of the research to ensure its accuracy and integrity.

## Guarantor

All authors read and approved the final manuscript, agreeing to take full responsibility for all aspects of the research to ensure its accuracy and integrity.

## Research registration number

Not available.

## Conflict of interest statement

The authors state that they have no conflict of interest to be mentioned.
